# Single-agent chemotherapy in low-risk gestational trophoblastic neoplasia

**DOI:** 10.22088/cjim.14.1.108

**Published:** 2023

**Authors:** Leila Mousavi Seresht, Marjaneh Farazestanian, Zohreh Yousefi

**Affiliations:** 1 Department of Gynecology and Oncology, Isfahan University of Medical Sciences, Isfahan, Iran; 2 Department of Gynecology and Oncology, Ghaem Hospital, Mashhad University of Medical Sciences, Mashhad, Iran

**Keywords:** Actinomycin, Gestational trophoblastic neoplasia, Methotrexate, Outcome, Single-agent chemotherapy

## Abstract

**Background:**

Low-risk gestational trophoblastic neoplasia could be cured in the case of appropriate management with single-agent chemotherapy. This study was carried out to compare the efficacy of single-dose methotrexate versus Actinomycin-D in low-risk gestational trophoblastic neoplasia to analyze the most effective agent.

**Methods:**

This retrospective cohort study was conducted on the medical record of 170 cases with the diagnosis of low-risk gestational trophoblastic neoplasia from 2012 to 2019 to evaluate the response rate of single-dose weekly-methotrexate versus biweekly-Actinomycin-D.

**Results:**

Single agent chemotherapy was required in 170 patients with final risk score of less than 7. Among the 100 cases under weekly-methotrexate therapy, 29 patients were required second-line chemotherapy with Actinomycin-D and combination therapy which means complete remission of 71% with methotrexate, in comparison with 78.5% in the other group. Resistance was mostly seen in patients with documented choriocarcinoma in histology who had not received timely diagnosis and treatment.

**Conclusion:**

Individualized decision in the management of low-risk gestational trophoblastic neoplasia cases, based on histology, HCG, and history is the corn stone in successful treatment.

Gestational trophoblastic neoplasia (GTN) defined as the malignant transformation of placenta in consequent of any type of anticidental pregnancy (1). Regarding to the metastatic behavior of this group of gestational neoplasia, patient prognosis is predicted using the International Federation of Gynecology and Obstetrics (FIGO) prognostic scoring system. GTN cases categoraized in to low-risk patients scoring 0–6 and are expected with low-risk of resistance to single agent chemotherapy, and patients scoring ≥ 7 are with high risk of drug resistance (2-4). Although single-agent chemotherapy (ChT) known as the initial treatment protocol in low-risk GTN, the preferred agent, based on toxicity, cost, and prognostic factors are controversial (5-7). Based on Mousavi et al.’s study, 44% of cases had not shown proper response by methotrexate (MTX) therapy, but this study revealed an even high rate of treatment failure, near 36%, in Actinomycin-D (ACT) (8). On the contrary, Mamour. G et al. believed in the superiority of MTX as a safe and available agent, especially in the developing countries (9). The present study sought to evaluate a considerable number of low-risk GTN cases to demonstrate more evidence-based data in this scientific challeng This retrospective study was conducted on the medical records of low-risk GTN cases from 2012 to 2019 at a tertiary referral institute in Iran. The study was approved by the Ethics Board Committee with this number of IR.MUI.MED.REC.1400.607

The diagnostic criteria for post-molar GTN were based on histology, and/ or plateau or raised level of β-Hcg titrate. The patient was staged by FIGO staging criteria and the oral contraceptive was prescribed in all. Among the 359 cases with GTD diagnosis, 185 patients had progressed to low-risk GTN. History of re-evacuation curettage or missing medical records was considered as the exclusion criterion. According to financial policies, weekly muscular-MTX in a dose of 50 mg/m^2^ was used in most of the cases, and bi-weekly ACT was preserved for patients who were not eligible for weekly monitoring with an estimated dose of 1.25 mg/m^2^. ChT was continued until achieving normal β-Hcg level and three more consolidation courses. The decision-making pathway is illuminated in Figure 1. Finally, the treatment outcome, drug resistance rate, median courses of ChT, and time of follow-up, and side effects were evaluated. The data analysis was carried out using SPSS statistical software (Version 23.0, SPSS, Inc., Chicago, IL). Additionally, treatment outcome by the type of regimen was assessed using multiple logistic regressions.Written informed consent was obtained from each patient for the publication of this study. 

## Results

170 patients with the diagnosis of low-risk GTN was enrolled in the study and there was no significant difference in age, parity and the Hcg level at the time of diagnosis. Among the included cases, 57 patients were categorized in Figo-stage of 2 or 3 due to the presence of adjacent organ invasion or lung metastasis. Their characteristic data are summarized in "table 1". 

Unfortunately during the administration time of MTX, 6 patients progressed to stage 4 and were required for more aggressive treatment with combination-ChT. So, the response rate of first-line MTX was estimated at 71%, in comparison with 78.5% with ACT. The overall response rate with single-agent ChT in low-risk GTN, as present in "table. 2’, was calculated in about 84.7%.

The most reported complications accompanied by MTX were conjunctivitis and liver toxicity, which lead to ChT regimen exchange in 6 cases. There was only one reported adverse effect with ACT with cutaneous necrosis in the site of infusion. The median interval between GTN diagnosis and the presence of chemo-resistance features were 6 to 7 weeks and the response was expected to be achieved after 6.35±2.87 courses of ChT. The calculated number of ChT courses to achieving remission in cases that need to be treated with second-line single-agent ChT was 13.46±4.36.

**Table 1:** Characteristic fining of low-risk GTN cases

**The characteristic data**		**N (%)**
Maternal age (year)		29.04±8.33^ƚ^	
Parity	0	19(10.9)
	1	59(34.5)
	2-3	82(48.2)
	>4	10(6.4)
Incidental Pathology in evacuation of molar pregnancy	Complete mole	120(70.6)
	Incomplete mole	27(15.7)
	Invasive mole	18(10.8)
	choriocarcinoma	5(2.9)
FIGO staging system	I	113(66.3)
	II (and score<7)	28(16.3)
	III (and score<7)	29(17.2)

ƚ mean, N; number

**Figure 1:**
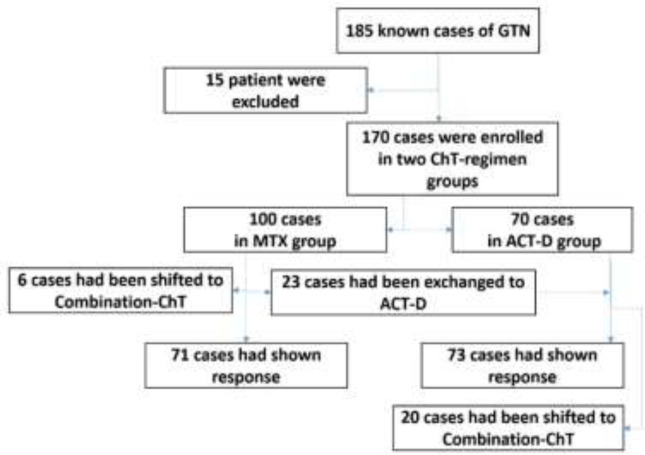
The pathway of study-designing. ACT; Actinomycin-D, ChT; Chemotherapy, GTN; Gestational trophoblastic neoplasia, MTX; methotrexate

As described in "figure. 1", Single-agent chemotherapy with MTX was prescribed in 100 cases and needs to be exchanged to ACT in 23 patients due to response failure or adverse effects. 

**Table 2:** The detailed illustration of response with the different regimens of chemotherapy in low-risk gestational trophoblastic cases

**Chemotherapeutic agent **		**N**
**MTX**	Total	100
	Response/ Non-response	71/29
**ACT**	Total	93
	Response/ Non-response	73/20
**EMA-Co**	Total	26
	Response/ Non-response	26/0
**EMA-EP**	Total	0
	Response/ Non-response	-
**MTX→ACT**		23
**ACT→EMA-CO**		7
**MTX→EMA-CO** **MTX→ACT→EMA-CO**		6 13
**EMA-Co →EMA-EP**		0

ACT; Actinomycin-D, EMA; Etoposide, Methotrexate, and Actinomycin-D, CO; Cyclophosphamide and Oncoverin, EP; Etoposide, Platinum. N; number of cases in each group.

## Discussion

Based on the evidence, the single-agent ChT, MTX vs ACT, is a legible approach in the management of low-risk GTN, but the best treatment option is still under consideration (10). In the latest Cochrane study, MTX was demonstrated as the most common agent for first-line single-agent ChT according to lower cost and lesser risk of complication in comparison with ACT (11, 12). The result of the present study was not in favor of further toxicity with ACT but supported its worse complication, like cutaneous necrosis, compared with reversible MTX-induced hepatic dysfunction. In addition to the remaining challenge on superior safety of MTX, the GOG-174 study disagreed with the cost-effectiveness of MTX, given that more courses were needed in resistance patients (4). 

Leaving aside the cost debate, the present report had supported the finding of the Osborne et al.’s trial that revealed the superiority of ACT, even though the MTX prescribed dose was higher in the present study (5). The other point is the probable effect of race on expected response, as the median duration of ChT in the present study on Asians to achieving response was 6.35±2.87 in comparison with reported response in other races following only 2 courses of ChT prescription (13). That could be another rational support for the superiority of ACT with documented less-needed cycles in this population. Above all, it must emphasize the essential role of experience and appropriate surveillance in obtaining the best result. Above all it must have emphasis on the need for further prospective study with precise study design to eliminate the chance of borderline risk score on the patient’s response which was a considerable limitation in the present study with retrospective nature.

In conclusion individualized decision in the management of low-risk gestational trophoblastic neoplasia cases, based on histology, HCG, and history is the corn stone in successful treatment.
